# Sequential emergence and clinical implications of viral mutants with K70E and K65R mutation in reverse transcriptase during prolonged tenofovir monotherapy in rhesus macaques with chronic RT-SHIV infection

**DOI:** 10.1186/1742-4690-4-25

**Published:** 2007-04-06

**Authors:** Koen KA Van Rompay, Jeffrey A Johnson, Emily J Blackwood, Raman P Singh, Jonathan Lipscomb, Timothy B Matthews, Marta L Marthas, Niels C Pedersen, Norbert Bischofberger, Walid Heneine, Thomas W North

**Affiliations:** 1 California National Primate Research Center, University of California, Davis, USA; 2 Division of HIV/AIDS Prevention, National Center for HIV, STD and Tuberculosis Prevention, Centers for Disease Control and Prevention, Atlanta, USA; 3 Center for Comparative Medicine, University of California, Davis, USA; 4 Department of Medicine and Epidemiology, School of Veterinary Medicine; University of California, Davis, USA; 5 Gilead Sciences, Foster City, USA; 6 Department of Molecular Biosciences, School of Veterinary Medicine, University of California, Davis, USA

## Abstract

**Background:**

We reported previously on the emergence and clinical implications of simian immunodeficiency virus (SIVmac251) mutants with a K65R mutation in reverse transcriptase (RT), and the role of CD8+ cell-mediated immune responses in suppressing viremia during tenofovir therapy. Because of significant sequence differences between SIV and HIV-1 RT that affect drug susceptibilities and mutational patterns, it is unclear to what extent findings with SIV can be extrapolated to HIV-1 RT. Accordingly, to model HIV-1 RT responses, 12 macaques were inoculated with RT-SHIV, a chimeric SIV containing HIV-1 RT, and started on prolonged tenofovir therapy 5 months later.

**Results:**

The early virologic response to tenofovir correlated with baseline viral RNA levels and expression of the MHC class I allele Mamu-A*01. For all animals, sensitive real-time PCR assays detected the transient emergence of K70E RT mutants within 4 weeks of therapy, which were then replaced by K65R mutants within 12 weeks of therapy. For most animals, the occurrence of these mutations preceded a partial rebound of plasma viremia to levels that remained on average 10-fold below baseline values. One animal eventually suppressed K65R viremia to undetectable levels for more than 4 years; sequential experiments using CD8+ cell depletion and tenofovir interruption demonstrated that both CD8+ cells and continued tenofovir therapy were required for sustained suppression of viremia.

**Conclusion:**

This is the first evidence that tenofovir therapy can select directly for K70E viral mutants *in vivo*. The observations on the clinical implications of the K65R RT-SHIV mutants were consistent with those of SIVmac251, and suggest that for persons infected with K65R HIV-1 both immune-mediated and drug-dependent antiviral activities play a role in controlling viremia. These findings suggest also that even in the presence of K65R virus, continuation of tenofovir treatment as part of HAART may be beneficial, particularly when assisted by antiviral immune responses.

## Background

Tenofovir (9-[2-(phosphonomethoxy)propyl]adenine; PMPA) is a commonly used antiretroviral compound which selects for the K65R mutation in reverse transcriptase (RT); this mutation is associated with a 2- to 5-fold reduced *in vitro *susceptibility to tenofovir [1,2]. Many tenofovir-containing regimens induce strong and long-lasting suppression of viremia in the majority of persons, with a low occurrence of the K65R mutation [1,3-5]; the emergence of K65R mutants in such patients was not always associated with a viral rebound [1,5,6]. However, a lower virologic success rate has been observed when tenofovir was used in specific combinations with other drugs with overlapping resistance profile (e.g., lamivudine, didanosine and abacavir), and the K65R mutation was found in approximately 50% of patients with a less-than-desired virologic response on such regimens [6-11].

Although much progress has been made [12], many unresolved questions remain regarding the exact virulence and clinical implications of drug-resistant viral mutants, and how to use this information to make treatment decisions. This is also true for K65R viral mutants. While the K65R mutation reduces replication fitness of HIV-1 *in vitro *relative to wild-type virus [13], it is unclear to which extent this can be extrapolated to virus replication fitness *in vivo*, especially when K65R is accompanied by other mutations in RT; some mutations may be compensatory (to improve replicative capacity), while the combination of K65R with certain other drug-selected mutations may be deleterious for viral replicative capacity (e.g., L74V, certain thymidine-analogue mutations), or may restore viral susceptibility to other compounds of the drug regimen [14-17]. It is also unclear whether the detection of K65R HIV-1 mutants is a valid criterion for withdrawing tenofovir from the patient's regimen, as it is possible that tenofovir still exerts some residual antiviral activity *in vivo *against replication of K65R HIV-1.

Simian immunodeficiency virus (SIV) infection of macaques has been a useful animal model to study the emergence, virulence and clinical implications of viral mutants during drug treatment [18]. Prolonged tenofovir monotherapy of macaques infected with virulent SIVmac251 resulted in the emergence of mutants with the K65R mutation in RT [19,20]. In the absence of tenofovir treatment, these K65R SIV isolates replicated *in vivo *to high levels and induced a disease course indistinguishable from that of wild-type virus [21]. In the presence of tenofovir treatment, however, disease-free survival was improved significantly, and some animals were able to suppress viremia of K65R virus to low or undetectable levels for 4 to more than 10 years [20-22]. Further experiments, using *in vivo *CD8+ cell depletions and treatment interruption, revealed that this suppression of K65R viremia depended on strong CD8+ cell-mediated immune responses, but that continued tenofovir therapy was also still necessary [20]. However, even when K65R viremia was not suppressed, continued tenofovir treatment was, surprisingly, associated with clinical benefits (i.e., disease-free survival) that were larger than predicted based on viral RNA levels and standard immune markers [22].

Because there are some important differences in the amino acid sequence of HIV-1 and SIV RT which affect susceptibilities and the mutational patterns to antiviral drugs [23], it is unclear to what extent these findings from the SIV model regarding the *in vivo *emergence, virulence and clinical implications of K65R viral mutants during tenofovir treatment can be extrapolated to HIV-1 RT. Some experimental procedures (such as CD8+ cell depletions, or prolonged tenofovir monotherapy), however, are not ethically or logistically feasible to study in HIV-1 infected humans. Because there is so far no optimal animal model that uses HIV-1, the currently best approach to unravel such questions about HIV-1 RT is the use of macaques infected with RT-SHIV, a chimeric virus consisting of SIVmac239 in which the RT gene is replaced by the counterpart of HIV-1 [24,25]. While RT-SHIV is virulent in macaques, the early studies (which used small animal numbers) found that viremia and the rate of disease progression were variable and on average lower than that observed with SIVmac239 or with other virulent SIV isolates, such as SIVmac251 [20,25-28]; this is likely because the insertion of a foreign RT into SIV affected its replicative ability [24]. Thus, a long-term study was performed to address the following questions through sequential experiments: (i) does *in vivo *passage of RT-SHIV lead to higher or more consistent virulence, (ii) does prolonged tenofovir treatment initiated during chronic RT-SHIV infection lead to the emergence of K65R viral mutants, (iii) what are the clinical implications of K65R mutants, and (iv) what is the role of CD8+ cells and continued tenofovir treatment in controlling viremia of K65R RT-SHIV?

The current report is the first one to demonstrate that during prolonged tenofovir therapy, RT-SHIV infected animals developed first K70E mutants, which were then replaced by K65R mutants. Further experiments in one animal that suppressed K65R viremia to undetectable levels demonstrated that, similarly to the findings in the SIVmac251 model, both CD8+ cell-mediated antiviral immune responses and continued tenofovir therapy were important to obtain maximal suppression of RT-SHIV viremia. This suggests that maintaining tenofovir as part of HAART, particularly when CD8+ cell-mediated immune responses are good and no better therapies are available, may still offer clinical benefits to persons infected with K65R mutants.

## Results

### *In vivo *passage of RT-SHIV and establishment of persistent infection

**Figure 1:**
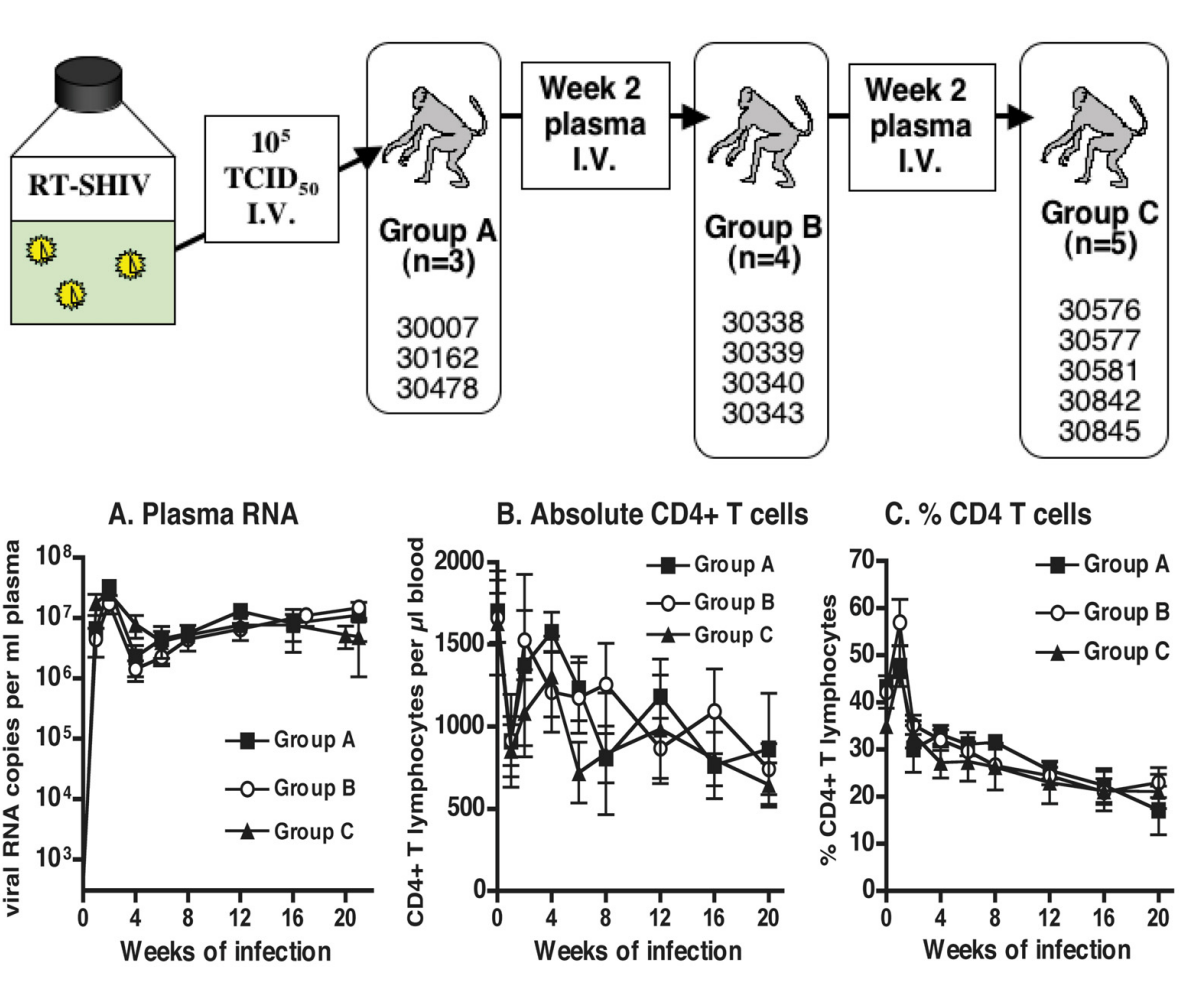
**Serial *in vivo *passage of RT-SHIV: effect on virulence**. A high dose of RT-SHIV (10^5 ^TCID_50_), propagated *in vitro *in CEMx174 cells, was inoculated intravenously in 3 animals (group A). Plasma collected 2 weeks later was pooled and administered intravenously to 4 animals (group B). The same procedure was repeated for the final passage into 5 animals (group C). There were no significant differences between the 3 groups with regard to viral RNA levels (calculated after log-transformation; graph A), mean absolute CD4+ T lymphocytes counts/μl and % CD4+ T lymphocytes in peripheral blood, (graphs B, C). Error bars indicate SEM.

**Figure 2:**
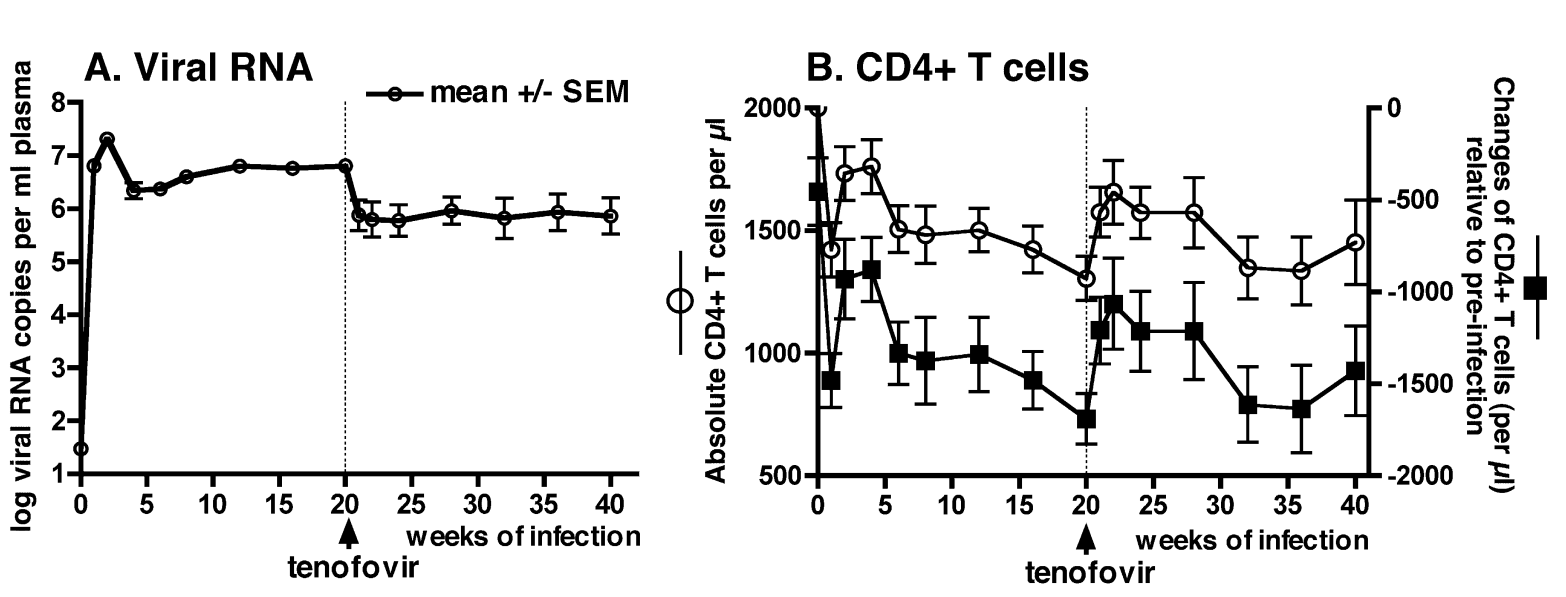
**Effect of tenofovir therapy on mean viral RNA levels and CD4+ T lymphocyte counts**. (A) Following tenofovir treatment (vertical dotted line), the average viremia (mean +/- SEM, calculated after log transformation) declined to approximately 1 log below pre-therapy baseline levels; note that the length of the SEM bars indicates larger variability of viremia after tenofovir therapy than before treatment (as shown in the individual graphs in figure 3). (B). The time course of CD4+CD3+ T lymphocyte counts in peripheral blood of the 12 animals is presented as absolute values (mean +/- SEM) along the left Y-axis; in addition, for each individual animal, the change in CD4+ T cell counts relative to its pre-infection value (time zero) was calculated, and the mean +/- SEM of these changes is presented along the right Y-axis. Both analyses gave (as expected) identical statistical conclusions.

Although the molecular clone of RT-SHIV is virulent in macaques, earlier studies found that infection resulted in a variable peak and set-point of viral RNA levels in plasma [24,26-28]. In an attempt to further increase its virulence, the cloned virus was subjected to 2 sequential *in vivo *passages (Fig. 1). A first group of 3 animals (group A) was inoculated intravenously with 10^5 ^TCID_50 _of *in vitro *propagated RT-SHIV. Plasma collected two weeks after infection was pooled and 0.6 ml of this pool (containing ~19 × 10^6 ^viral RNA copies; ~1,400 TCID_50_) was administered intravenously to a second group of 4 animals (Fig. 1, group B). The same procedure was repeated, and 0.6 ml pooled plasma collected from group B animals at 2 weeks of infection (~10 × 10^6 ^viral RNA copies; ~1,000 TCID_50_) was injected intravenously into 5 animals (Fig. 1, group C). Peak virus levels for animals of all 3 groups were observed at 1 to 2 weeks after infection and ranged from 9 to 43 million copies RNA per ml plasma (Fig. 1A), and 2,200 to 32,000 TCID_50 _per million PBMC (data not shown). The rapid serial passage in macaques did not have any detectable effect. The 3 animal groups had similar viral RNA levels in plasma and infectious titers in PBMC, and a similar decline in absolute counts and percentages of CD4+ T lymphocytes and CD4+/CD8+ T cell ratios during the first 20 weeks of infection (two-way ANOVA: p values of passage effect >0.05; Fig. 1). During the first 20 weeks of infection, all 12 animals had a decrease in absolute CD4+ T cell counts (mean loss of 927 (range 480–1590) cells per μl; Fig. 2B); this meant a median decrease of 55% (range 28–83%) of their absolute CD4+ T cell counts. All 12 animals mounted strong humoral immune responses to SIV, as the SIV-specific IgG titers in plasma (measured by ELISA) were > 102,400 by eight weeks of infection (data not shown). There was no detectable difference among the three groups in response to subsequent tenofovir treatment and disease-free survival, and accordingly the groups are combined for the presentation of the remainder of the study.

### Tenofovir monotherapy of RT-SHIV infected macaques: early virologic and immunologic responses

**Figure 3:**
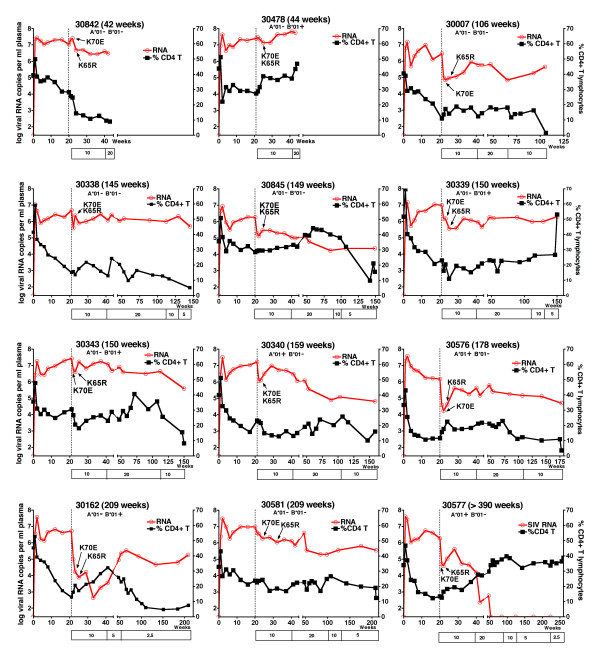
**Individual data of plasma viral RNA levels and percentages of CD4+ T lymphocytes**. Twelve RT-SHIV infected juvenile macaques were started on tenofovir treatment (10 mg/kg subcutaneously, once daily) at approximately 20 weeks of infection (vertical dotted line). Changes in tenofovir dosage regimens (in mg/kg) are indicated in the boxes along the X-axis. Viral RNA levels in plasma (in log-transformed copy number per ml plasma) are presented along the left Y-axis, while the % CD4+ T lymphocytes in peripheral blood is presented along the right Y-axis. The earliest detection of the K70E or K65R mutation in viral RNA in plasma virus by real-time RT-PCR is indicated (see Figure 6 for more details). Animals are arranged according to disease-free survival (which is indicated after each animal number). The presence or absence of the expression of the MHC I alleles Mamu-A*01 and Mamu-B*01 is indicated below each animal number.

Untreated RT-SHIV infected macaques have generally little change in viremia once a viral set-point is established after ~8 to 12 weeks of infection [25,26,29]. In the current study, the 12 RT-SHIV animals were started on tenofovir monotherapy (10 mg/kg, subcutaneously once daily) at approximately 20 weeks of infection. This starting dose was selected because it is pharmacokinetically similar (based on plasma AUC levels of ~20 μg.h/ml) to the intravenous tenofovir regimen of the initial human clinical trials [30]. Tenofovir treatment was associated with an average 10-fold decrease in viral RNA levels after 1 week of treatment (Fig. 2A). However, there was much individual variability; 10 animals had a decrease in plasma viral RNA levels (mean decrease: 21-fold; range: 2 to 53-fold), while the remaining 2 animals (numbers 30842 and 30478; Fig. 3) had no decrease after 1 week of treatment. Infectious virus titers in PBMC showed similar patterns as the plasma viral RNA levels (data not shown). The early effect of tenofovir therapy on the percentage of CD4+ T lymphocytes in peripheral blood was variable, as only half of the animals showed a relative increase of ≥ 3% within 2 weeks of therapy (Fig. 3). However, relative to the baseline value at the onset of tenofovir therapy, after 2 weeks of treatment all 12 animals had an increase in total lymphocyte counts (median increase of 51% (range 22–272%; p = 0.001, two-tailed paired t test), and 11 animals had an increase in absolute CD4+ T cell counts (mean change of + 469 (range from -149 to +1291) cells per μl; p = 0.002, two-tailed paired t test; Fig. 2B), which meant a median increase in absolute CD4+ T cell counts of 71% (range of relative change: -21 to +183%). This significant increase in absolute CD4+ T cell counts was transient, as values returned to pre-therapy baseline values after 12 weeks of tenofovir therapy (32 weeks of infection; Fig. 2B; two-tailed paired t test p values ≥ 0.05). Absolute CD4+ T cell counts then stabilized for most animals until they declined concomitantly with the development of clinical disease symptoms.

**Figure 4:**
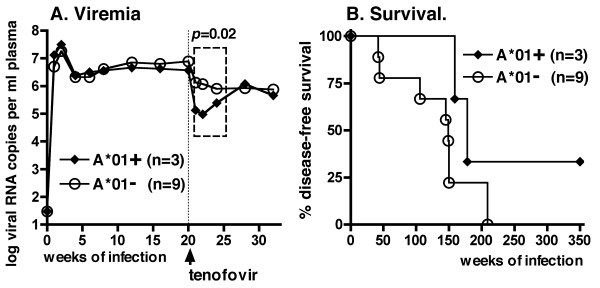
Association of expression of MHC class I allele Mamu-A*01 with viremia and early virologic response to tenofovir therapy. (A) No significant difference was detected between the 3 Mamu-A*01-positive and the 9 Mamu-A*01-negative animals with regard to viremia during the first 20 weeks of infection (two-way ANOVA, p = 0.86) or virus levels at the start of tenofovir treatment (vertical dotted line; two-tailed t-test: p = 0.29). However, during the first 4 weeks following the start of tenofovir treatment (dashed-line box), Mamu-A*01-positive animals had a bigger reduction in viral RNA levels than Mamu-A*01-negative animals (two-way ANOVA, p = 0.02); there was no association of the Mamu-B*01 allele with viremia (data not shown). (B) Comparison of disease-free survival following tenofovir treatment revealed no significant difference between the 3 Mamu-A*01-positive and 9 negative animals (logrank test, p = 0.14).

Three of the 12 animals expressed the major histocompatibility complex (MHC) class I allele Mamu-A*01; 4 other animals expressed the MHC class I Mamu-B*01 allele. Although there was no significant effect of the presence of either one of these alleles and viremia during the first 20 weeks of infection (prior to tenofovir therapy), Mamu-A*01-positive animals responded initially to tenofovir therapy with lower viral RNA levels than Mamu-A*01-negative animals (first 4 weeks of treatment, two-way ANOVA, effect of Mamu-A*01 p = 0.02; Fig. 4A). But between 8 to 20 weeks of tenofovir treatment (i.e., 28 to 40 weeks of infection), concomitant with the detection of viral mutants (see below), there was no significant difference in viremia between Mamu-A*01-positive and -negative animals anymore (two-way ANOVA, p = 0.46).

**Figure 5:**
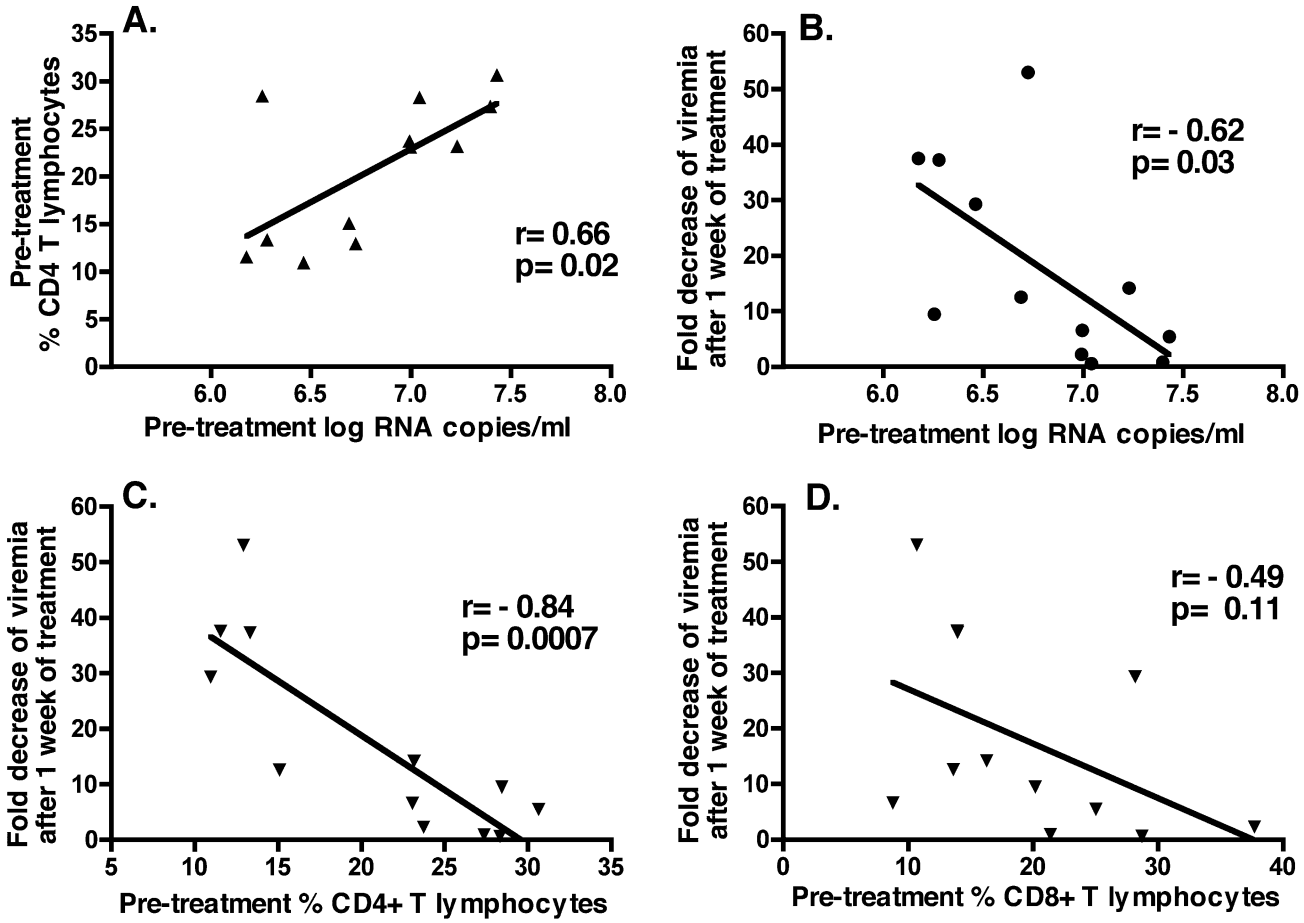
**Correlations of baseline viral and immunologic parameters and early virologic response to tenofovir therapy**. Pre-treatment values of viral and immunologic parameters are baseline values at the onset of tenofovir treatment (i.e., ~20 weeks of infection). The early virologic response is expressed as fold decrease of viremia (viral RNA levels in plasma) after 1 week of tenofovir therapy. Spearman r and two-tailed p values are indicated for each graph. The pre-treatment viral RNA level correlated with the pre-treatment % CD4+ T lymphocytes (graph A), but did not correlate significantly with percentages of CD8+CD3+ T lymphocytes or CD20+ B lymphocytes (p = 0.40 and 0.12, respectively; data not shown). The early virologic response had significant correlations (p ≤ 0.05) with the pre-treatment viral RNA levels (graph B), % CD4+ T lymphocytes (graph C), and percentage and absolute counts of CD20+ B lymphocytes (data not shown). There was no correlation between the early virologic response to tenofovir and baseline lymphocyte counts, the percentages and absolute counts of CD3-CD8+ NK cells in peripheral blood, or SIV-specific IgG titers in plasma (data not shown).

We examined whether other baseline markers at the onset of tenofovir therapy were predictive of the early virologic response. The magnitude of the early virologic response (i.e., fold decrease of viremia after 1 week of treatment) correlated negatively with baseline viral RNA levels (Pearson r = -0.62, two-tailed p = 0.03; Fig. 5B), and negatively with baseline % CD4+ T lymphocytes (Pearson r = -0.84; two-tailed p = 0.0007; Fig. 5C), but not with % CD8+ T lymphocytes (p = 0.11; Fig. 5D). Baseline viral RNA correlated positively with % CD4+ T lymphocytes (Pearson r = 0.66; two-tailed p = 0.019; Fig. 5A).

### Selection of K70E followed by K65R mutation in RT during prolonged tenofovir monotherapy

**Figure 6:**
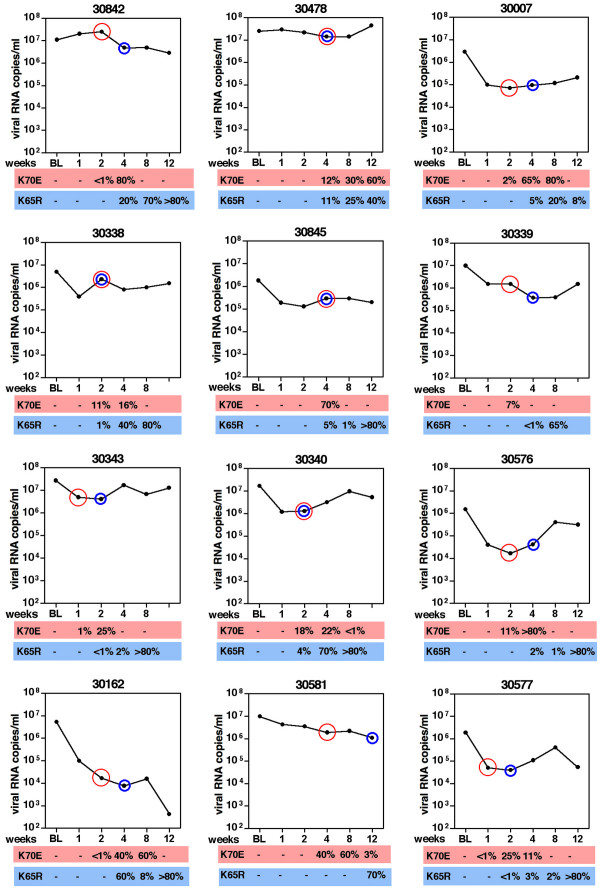
**Kinetics of K70E and K65R RT mutants during tenofovir therapy**. Twelve RT-SHIV infected macaques were started on tenofovir treatment 5 months after infection. Real-time PCR technology was used to quantitate K65R and K70E RT mutants in plasma samples; values are expressed as percentage of total viral RNA copy number. At the onset of tenofovir therapy (i.e, baseline, BL), no K65R and K70E virus could be detected. The red and blue circles indicate the first detection of K70E and K65R, respectively; weeks indicate weeks of tenofovir treatment.

**Figure 7:**
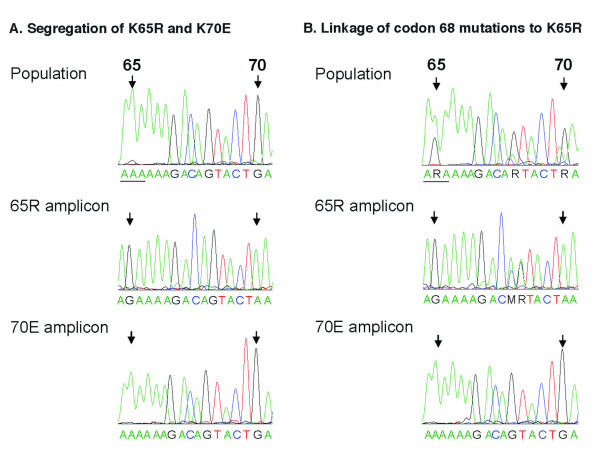
**Segregation of K65R and K70E mutations, and linkage of codon 68 mutations with K65R**. Plasma viral RNA samples in which real-time PCR assays detected both K65R and K70E mutations were analyzed further; representative samples are shown. Panel A: animal 30007, week 8 of tenofovir treatment (see Figure 6). Population sequencing revealed a mixture of wild-type and mutant variants at both codons 65 and 70 (top graph); the bar indicates the codon reading frame. The selective amplification of virus sequences containing 65R or 70E by real-time PCR allowed for their enrichment from the virus background quasispecies. Direct sequencing of the mutation-specific amplicons revealed that the 65R amplicon (AGA, arginine) had wild-type sequence at codon 70 (AAA, lysine; middle graph), while the 70E amplicon (GAA, glutamic acid) had wild-type at codon 65 (lysine, AAA; bottom graph). Thus, the K65R and K70E mutations were on separate viral genomes. Panel B: animal 30478, week 12 of tenofovir treatment. The mutation-specific amplicons from this specimen also exhibited segregation of K65R and K70E. The sequence of the 65R amplicon demonstrated mutations at codon 68 (middle graph), while the 70E amplicon had wild-type sequence (AGT, serine) at codon 68 (bottom graph). The presence of mixtures is indicated (M is A or C; R is A or G).

For 9 of the 10 animals for which viremia decreased following the onset of tenofovir therapy, the nadir of plasma viral RNA levels was reached after 2 to 4 weeks of treatment (Fig. 3). Subsequently, there was a partial rebound of viremia, although the average virus levels remained approximately 10-fold below the baseline levels (i.e., at the onset of tenofovir therapy; Fig. 2A). This rebound was associated with the detection of RT mutations that were not detectable prior to tenofovir treatment. Population sequencing of virus isolates from PBMC revealed that the 2 most frequent mutations that emerged sequentially early after tenofovir therapy were a lysine to glutamic acid mutation at codon 70 (K70E; AAA to GAA) followed by the K65R mutation (AAA to AGA)(table 1). Therefore, more sensitive real-time PCR assays were developed to detect and quantify these 2 mutants in viral RNA in sequential plasma samples. While population genotyping of DNA from PBMC-derived virus isolates detected K70E mutants in only 10 animals, the real-time PCR method detected K70E mutants in plasma RNA of all 12 animals within 1 to 4 weeks (median 2 weeks) of tenofovir treatment (Fig. 3, 6). For all 12 animals, the K65R mutation became detectable in plasma viral RNA within 2 to 12 weeks of treatment (median time, 4 weeks). Due to its high sensitivity for detecting low-frequency mutants, the real-time PCR assay detected the K65R mutation prior to its detection by population genotyping in 11 animals (table 1). When both K65R and K70E were detected in plasma viral RNA samples, direct sequencing of the mutation-specific real-time PCR amplicons demonstrated that the 2 mutations were on separate genomes (Fig. 7A). By 12 weeks of treatment, K70E became undetectable prior to or coinciding with the establishment of the K65R mutation in 10 of the 12 animals (Fig. 6).

**Table 1:** Mutations in RT detected in virus isolated from RT-SHIV infected macaques.

**Animal number**	**Time of Infection **(weeks)	**Codon 65 mutation**	**Codon 70 mutation**	**Other RT mutations**
**30007**	21 (Tx)	-	-	V75L, G196R, M357T/M
	23	-	-	V75L, G196R
	25	-	K70E	G196R, L214F, M357T
	29	K65R/K	-	G196R, L214F, M357T
	33	K65R	-	G196R, L214F
	41	K65R	-	S68G, P150S, E194K, G196R, I202V, L214F
	65	K65R	K70N	S68G, A98G, Y115F
	93	K65R	K70H	V8I, S68G, A98G, Y115F, K154E, A158P, I159L, G196R, L214F, D218E, K219R, H221P
	115	K65R	K70H	V8I, K45Q, S68G, A98G, Y115F, V179I, G196R, L214F, K219R, K275R, R277K, M357T
**30162**	21 (Tx)	-	-	V75L, G196R, K275R
	25	-	-	V75L, G196R, K275K/R
	29	-	-	V75L, G196R, K275R
	33	-	-	V75L, G196R, K275R
	37	-	-	K22R, W88S, L214F
	41	K65R	-	W88S, Y115F, E194K, L214F
	65	K65R	-	S68S/N, W88S, Y115F
	93	K65R	K70T	S68G, K70T, W88S, Y115F, K154E, A158P, L214F, K219Q
	209	K65R	K70T	S68G, K70T, W88S, Y115F, T139A, I178M, L214F, H221Y, K275R, R277K, M357N
**30478**	21 (Tx)	-	-	V75L, H208L, L214F
	25	-	-	V75V/L, H208L. L214F
	29	-	K70E/K	H208L, L214F
	33	K65K/R	K70E/Q/K	G196R, L214F
	37	K65R	-	S68N, G196R, L214F
	41	K65R	-	S68N, G196R, L214F
**30338**	21 (Tx)	-	-	G196R
	22	-	-	V75L, G196R, L214F, K275R, M357T
	23	-	-	V21I, V75L, G196R, L214F
	25	-	K70K/E	V75L, G196R, L214F
	29	K65R	-	G196R, L214F, M357T
	33	K65R	-	G196R, L214F, K275R, M357T
	41	K65R	-	S68N, Y115F, G196R, L214F
	59	K65R	K70Q	S68N, Y115F
	89	K65R	K70Q	K20R, Y115F, K154Q, A158T, I178M, E194K, G196R, L214F, K219Q
	145	K65R	K70Q	V8I, K20R, M41L, S68G, W88S, Y115F, F116W, I178M, G196R, L214F, H221Y, K275R, R277K, P294Q, M357T
**30339**	21 (Tx)	-	-	E194K, G196R,
	25	WT	-	W88S, G196R, L214F, M357T
	29	K65R	-	W88S, G196R, K275R, R277K, M357T
	33	K65R	-	S68R, W88S, G196R, L214F, K275R
	41	K65R	-	S68K, W88S, G196R, R199M, K219E
	59	K65R	-	S68K, W88S, Y115F, K219E
	89	K65R	-	K22R, K64R, S68K, W88S, Y115F, K154Q, A158P, I178M, G196R
	150	K65R	-	T39A, K45Q, K64R, S68K, W88S, Y115F, I178M, V195L, G196K, K219G, H221Y, K275R, R277K, M357T
**30340**	21 (Tx)	-	-	V75L, E194K, G196R,
	22	-	-	V75L, G196R
	23	-	K70K/E	G196R
	25	-	K70K/E	G196R
	29	K65R	-	G196R
	33	K65R	-	G196R, L214F
	41	K65R	-	S68G, Y115F, V118I, E194K, G196R, R199I
	89	K65R	-	K20R, S68G, W88S, Y115F, G196R, R199I, L214F, H221Y
	159	K65R	K70Q	S68K, W88S, Y115F, F116W, G196R, L214F, H221Y, S251N, R277K, M357T
**30343**	21 (Tx)	-	-	G196R, K219N
	22	-	-	G196R, K219N, K275R, M357T
	23	-	-	G196R, K275R, M357T
	25	K65K/R	K70K/E	G196R, K275R, M357T
	41	K65R	-	S68N, G196R
	59	K65R	-	S68N, Y115F, Y181C, K219N/D
	89	K65R	-	S68N, W88S, Y115F, F116W, G196R, K219H
	150	K65R	K70H	M41L, S68K, W88S, Y115F, F116W, V118I, I178M, G196R, K219H, K275R, R277K, M357T
**30576**	20 (Tx)	-	-	V75I, E194K, G196R, L210V, L214F
	21	-	-	V75L, G196R, L214F, K275R, G359S
	22	-	K70E/K	G196R, L214F
	24	-	K70E/K	G196R, E203G, L214F
	28	-	K70E/K	G196R, L214F, M357T
	32	K65R		S68N, G196R, L214F, K275R, M357T
	36	K65R		S68N, G196R, L214F, M357T
	40	K65R	-	S68N, I195T, G196R, L214F
	53	K65R	-	S68N, Y115F
	84	K65R	K70N	V8I, S68G, Y115F, F116W, Q145H, P150S, G196R, H208Q, L214F
	178	K65R	K70H	V7I, K45Q, S68G, Y115F, F116W, R172S, K173Q, I178M, G196R, I202V, L214F, K219R, K275R, R277K, M357T
**30577**	20 (Tx)	-	-	E194K, G196R
	22	-	-	V75L, G196R, L214F
	24	-	K70E/K	I178M, G196R, L214F
	28	K65K/R	K70E/K	I178M, G196R
	40	K65R	-	K20R, S68N, E194K, G196R, R199K, L210V, L214F
	47	K65R	-	S68N, G196R
	265 (no CD8)	K65R	-	K20R, S68N, G196R, L214F, Q248N
	296 (no Tx)	K65R	-	K20R, S68N, G196R, L214F, Q248N
**30581**	20 (Tx)	-	-	V75I, E194K, G196R,
	22	-	-	V75L, G196R, L214L/F
	24	-	K70K/E	V75L, G196R
	28	-	K70E	G196R, M357T
	32	K65R	-	G196R, L214F, M357T
	40	K65R	-	S68G, G196R, L214F
	53	K65R	K70T	S68G, F116W
	84	K65R	K70T	S68G, A98G, F116W, P150S, I159V, R172I, V179G, Q222L
	209	K65R	K70T	E40Q, K45Q, S68G, T69I, A98G, F116W, I178M, G196R, K219R, K275R, R277K, M357S
**30842**	20 (Tx)	-	-	V75L, E194K, G196R,
	22	-	-	V75L, G196R, L214F
	24	-	K70E	G196R, L214F
	28	K65R/K	-	G196R, L214F
	32	K65R	-	S68N, G196R, L214F, N218E, M357T
	40	K65R	-	E194K, G196R, L214F
	42	K65R	-	Y115F, Y181C, K219E
**30845**	20 (Tx)	-	-	V75I, E169K, E194K, G196R,
	21	-	-	V75V/L, G196R
	22	-	-	T69N/T, W88S, G196R, L214F
	24	-	K70K/E	G196R, K275R
	28	K65K/R	K70E	G196R
	32	K65R		G196R, L214F, K275R, M357T
	36	K65R		S68G, W88S, G196R, M357T
	40	K65R	-	S68G, W88S, E194K, G196R, L214F
	53	K65R	-	S68G, W88S, Y115F
	84	K65R	K70N	S68G, W88S, A98G, Y115F, P150S, D177N
	149	K65R	K70N	K11N, V21I, K22R, M41L, S68G, W88S, Y115F, F116W, V118I, H221Y, V245M, K275R, R277K, I275R, M357T

All data were obtained from PBMC isolates by population sequencing methods. (Tx) indicates the start of tenofovir therapy. (no CD8) and (no Tx) indicate the viral rebound during CD8+ cell depletion experiment and tenofovir withdrawal experiment of animal 30577, respectively.

The K65R mutation resulted in approximately 5-fold reduced *in vitro *susceptibility to tenofovir (data not shown). Other RT mutations, which were likely compensatory mutations, were also detected in viruses by population sequencing (table 1). Some mutations (e.g. V75I/L, E194K, G196R, L214F) were already present in some viruses obtained prior to tenofovir therapy, and most have previously been described in RT-SHIV isolates obtained from untreated macaques [25,31-33]. The mutations most commonly observed (sometimes transiently) after the detection of K65R included K20R (3 animals), M41L (3 animals), S68G/K/N (12 animals), K70H/N/T/Q (9 animals), W88S (6 animals), Y115F (9 animals), F116W (6 animals), V118I (3 animals), I178M (6 animals), L214F (11 animals), and K219Q/R/E/N/D/H/G (7 animals) (table 1). Sequencing of mutation-specific amplicons revealed that the codon 68 mutations were associated with K65R sequences and not K70E (Fig. 7B); the codon 68 mutations may thus represent mutations that compensate for the replicative fitness cost of K65R, as has been suggested for HIV-1 [5,34]. There was no obvious causative association between these additional RT mutations and the rate of disease progression. Instead, animals that had persistent viremia and longer survival accumulated more mutations in RT than animals that had a more rapid disease course; in other words, these additional mutations were not required for a relatively rapid disease course. The tenofovir regimen was increased for most animals at 40 weeks of infection from 10 to 20 mg/kg to determine if higher drug levels would reduce viremia or select for other patterns of RT mutations that have previously been reported to give higher levels of *in vitro *resistance to tenofovir, such as T69S-insertion mutations [35]. A pharmacokinetic study showed that the subcutaneous 20 mg/kg tenofovir regimen in this study gave plasma AUC levels (mean +/- SD: 27.6 +/- 6.7 μg.h/ml; range 18.7 to 39.2 μg.h/ml) slightly higher than those observed in the human trials with intravenous tenofovir dosing (22.5 +/- 9.8 μg.h/ml; [30]). This higher dosage regimen did not result in any consistent changes in viremia or any detectable changes in drug resistance patterns (Fig. 3; table 1). Instead, the onset of glucosuria and hypophosphatemia, signs indicative of renal toxicity associated with high-dose tenofovir regimens [36], necessitated a reduction of the individual dosage regimens to safer low-dose maintenance regimens (Fig. 3).

The median disease-free survival of the tenofovir-treated animals was 150 weeks (~3 years). With the caveat that animal numbers per group were low, there was no significant difference in disease-free survival between Mamu-A*01-positive and -negative animals (logrank test, p = 0.14; Fig. 4B). The two animals (animals 30842 and 30478) that did not have a reduction in viremia after the start of tenofovir treatment developed life-threatening immunodeficiency the earliest, at ~8–9 months of infection (Fig. 3). Nine chronically treated animals developed fatal disease after 2 to 4 years of infection. For these 11 animals, the gross and histopathologic changes (including lymphoid hyperplasia, lymphoid depletion and opportunistic infections such as Cryptosporidium or Pneumocystis *carinii*) were characteristic of terminal SIV-induced immunodeficiency. The remaining animal, number 30577, became a long-term survivor with undetectable viremia, even though its virus had the K65R mutation in RT. Therefore, this animal is described subsequently in more detail.

### The role of both CD8+ cells and tenofovir treatment in suppression of viremia of mutant viruses

**Figure 8:**
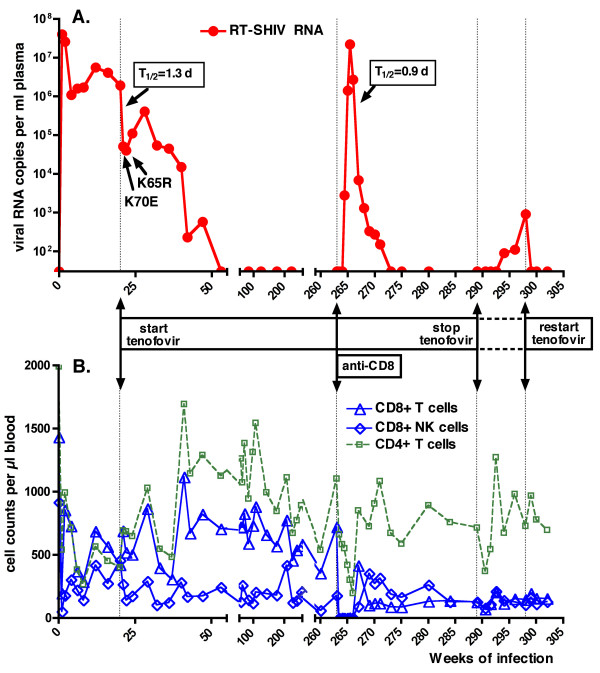
**Importance of both CD8+ cell-mediated immune responses and continuous tenofovir treatment in RT-SHIV infected animal 30577**. As indicated in Figure 3, animal 30577 was inoculated with RT-SHIV (time zero). Panel A and B represent viral RNA levels in plasma, and cell counts in peripheral blood (as measured by flow cytometry), respectively. Tenofovir treatment was started at 20 weeks of infection (vertical dotted line), resulting in an initial rapid 47-fold reduction of viremia (with estimated half-life of productively infected cells of 1.3 days). Despite an initial rebound associated with emergence of K70E followed by K65R viral mutants (table 1; Fig. 6), viremia became undetectable at 55 weeks of infection. At 264 weeks of infection, CD8+ cells were depleted using administration of 3 doses of cM-T807, while tenofovir treatment was continued (at a maintenance regimen of 2.5 mg/kg once daily). After a ~6 log increase in viremia (consisting of K65R virus), virus levels decreased rapidly (with estimated half-life of productively infected cells of 0.9 days) as soon as CD8+ cells started to return. At 289 weeks of infection, tenofovir treatment was interrupted for 9 weeks, and when viremia increased, restarted at the same regimen. The increased viremia during both experimental manipulations demonstrate that both CD8+ cells and continued tenofovir therapy were required for optimal suppression of viremia.

Before the start of treatment, animal 30577 had a viral set-point of ~10^6 ^viral RNA copies per ml plasma, and had the expected changes associated with a virulent infection, namely gradual decreases in percentages CD4+ T lymphocyte counts (< 15%; Fig. 3), absolute CD4+ T lymphocyte counts (< 500 per μl), and CD4+/CD8+ T lymphocyte ratios (ratio < 1 from week 8 to week 20). Thus, prior to tenofovir treatment, this animal was indistinguishable from the other RT-SHIV infected animals of this study. Following the onset of tenofovir treatment (at 20 weeks of infection), this animal had a rapid reduction in viremia from 1.9 million to 51,000 viral RNA copies/ml within one week; these kinetics suggest a half-life of productively infected cells of 1.3 days, very similar to our previous observations in SIVmac251-infected macaques receiving tenofovir treatment during acute viremia [20]. Coinciding with the detection of K70E and K65R mutants (Fig. 3, 8), plasma viremia rebounded from 40,000 (after 2 weeks of treatment) to 410,000 copies per ml at 8 weeks of treatment (i.e., 28 weeks of infection), but then gradually declined again and became undetectable (< 30 viral RNA copies/ml) from 53 weeks of infection onwards (i.e., 33 weeks of tenofovir treatment; Fig. 8). During continued tenofovir treatment, there was a gradual increase of CD4+ T lymphocyte values to normal pre-infection levels (percentage of CD4+ T lymphocytes: 30–39%; CD4+/CD8+ T-cell ratio 1.25–1.75; absolute CD4+ T lymphocyte counts: ≥ 700 per μl; Fig. 3, 8).

Similarly to our previous studies in tenofovir-treated SIVmac251-infected macaques [20], we investigated if this suppressed viremia of K65R virus in animal 30577 during prolonged tenofovir treatment was due to (i) a replication-impaired phenotype of the K65R mutant in this animal, (ii) strong CD8+ cell-mediated antiviral immune responses, and/or (iii) residual antiviral activity of the tenofovir regimen. Accordingly, 2 sequential experiments were performed, in which either CD8+ cells or tenofovir treatment were removed. In the first experiment, CD8+ cells were temporarily depleted via administration of 3 doses of the anti-CD8 antibody cM-T807 at 263 weeks of infection (~5 years of infection, and 4 years of undetectable viremia). Tenofovir treatment was continued at a stable maintenance regimen (2.5 mg/kg once daily, subcutaneously) during this period. Following the first dose of cM-T807, CD8+ T cells and NK cells were undetectable or very low (< 1% of lymphocytes; < 5 cells per μl) in peripheral blood for 3 weeks. Viral RNA levels became again detectable in plasma 10 days after the first cM-T807 injection, and peaked to 22 million RNA copies/ml on day 17 (Fig. 8). This dramatic increase in viral RNA levels in plasma was accompanied by an increase in infectious virus titers in PBMC, from undetectable (< 1) to 3,160 TCID_50 _per million PBMC on day 17 (data not shown). Real-time RT-PCR revealed that the plasma viral RNA at peak viremia consisted exclusively of K65R viral mutants, with no detection of the K70E mutation or wild-type sequence; this plasma viral RNA had also the S68N and G196R mutations; virus isolated from PBMC at peak viremia also had the expected K65R mutation, with relatively few other RT mutations compared to virus isolated 4 years earlier (table 1). When the CD8+ T lymphocytes and NK cells returned, plasma viral RNA levels and cell-associated infectious virus levels declined rapidly to undetectable baseline levels (< 30 RNA copies per ml plasma and < 1 TCID_50 _per million PBMC, respectively). The initial phase of very rapid decline of viral RNA levels during the return of CD8+ cells indicates a half-life of productively infected cells of 0.9 days, suggesting high antiviral potency of the returning CD8+ cells (Fig. 8). This transient rebound in viremia following CD8+ cell depletion was associated with a progressive decrease in CD4+ T lymphocyte counts (nadir of 199 cells per μl), which returned to normal levels (> 500 per μl) upon the reduction of viremia to undetectable levels (Fig. 8).

To determine if continued tenofovir treatment was required to maintain undetectable viremia in animal 30577, tenofovir treatment was interrupted at 289 weeks (~5.5 years) of RT-SHIV infection. Five weeks later, virus could be isolated again from PBMC, and this virus had the K65R and the same other RT mutations (including S68N) that were also detected during the viral rebound of the CD8+ depletion experiment (table 1). Starting 5 weeks after tenofovir withdrawal, viral RNA levels in plasma also became detectable again and increased slowly (Fig. 8). Real-time PCR performed on the plasma sample collected 9 weeks after tenofovir withdrawal (which had a viral load of 910 RNA copies per ml) revealed K70E but no K65R. Further sequencing of the plasma RNA revealed that this K70E virus had also G196R, but not S68N; although it did not have I178M, this K70E virus therefore resembled the virus that was detected early after the start of tenofovir treatment (see table 1, week 24 isolate). Nine weeks after tenofovir withdrawal (when viremia was 910 RNA copies/ml), tenofovir was restarted at the same regimen; both the PBMC-associated infectious virus levels and the plasma viral RNA levels returned to persistently undetectable levels (< 30 copies/ml) within one week of treatment (Fig. 8). Thus, continued tenofovir therapy was required to maintain optimal suppression of K65R and K70E viremia in this animal.

## Discussion

The current report provides further insights into the many aspects of chronic tenofovir therapy, including the sequential emergence and implications of K70E and K65R viral mutants. These data are important and timely, considering (i) the increased use of tenofovir in HAART regimens, and (ii) the ongoing clinical trials which investigate if chronic administration of tenofovir can protect high-risk groups against HIV infection, particularly since no prophylactic strategy is likely to be 100% effective [37]. The present data largely confirm the observations made previously with tenofovir in the SIVmac251 model [20], but the use of RT-SHIV led to novel findings, such as the transient detection of K70E viral mutants early after the start of tenofovir therapy. The advantage of an animal model is that it allows the control of many variables and experimental procedures (such as monotherapy and CD8+ cell depletions) that enable the study of mechanisms that would otherwise be difficult to unravel, and that are relevant to the clinical use of tenofovir-containing regimens in HIV-1 infected humans.

In the current study, intravenous inoculation of the first group of macaques with a high dose of RT-SHIV led to persistent viremia with set-point of 10^6 ^to 10^7 ^viral RNA copies per ml plasma, higher than that observed in some previous studies that used a lower virus inoculum [26,28,31]. Sequential *in vivo *passages of RT-SHIV did not lead to detectable changes in virulence, as determined by viremia, and CD4+ and CD8+ T lymphocyte counts. It is plausible that after the high-dose intravenous inoculation, the potential virulence was already maximized, as viremia levels were similar to those commonly observed with the parental SIVmac239 virus [38,39].

Others have reported that when RT-SHIV infected macaques were started on short-term tenofovir treatment (30 mg/kg, subcutaneously SID) either during acute viremia or during chronic infection (when viral set-points were 10^4 ^to 10^6 ^RNA copies/ml), viremia was rapidly reduced in all animals [26,29]. In the current study, the early virologic response to tenofovir was more variable, possibly because of the lower tenofovir dose (10 mg/kg) and the higher pre-therapy viral RNA set-points (~10^6 ^to 10^7 ^RNA copies/ml). In the present study, a higher pre-therapy viral RNA set-point (an indirect measure of weaker antiviral immune responses at the onset of treatment) correlated with a reduced early virologic response to tenofovir.

The expression of the MHC Class I allele Mamu-A*01 has previously been associated with a better immunologic control of the parental SIVmac239 virus, and antiviral CD8+ CTL responses directed against Mamu-A*01-restricted epitopes (including in Gag and Tat) were found to be dominant during SIVmac239 infection [40,41]. In the current RT-SHIV study, which had the limitation of small animal groups, there was no difference in viremia between Mamu-A*01 positive and -negative animals before the onset of tenofovir therapy. However, animals which expressed the Mamu-A*01 allele had a more pronounced drop in viremia during the first 4 weeks of tenofovir therapy than Mamu-A*01-negative animals. This observation suggests that any role of Mamu-A*01-dependent antiviral immune responses in attempting to control viremia was transiently unmasked or rescued by the concomitant tenofovir treatment. These findings are consistent with previous observations (including from studies that used CD8+ cell depletion) that demonstrated that the early virologic response to tenofovir and other drugs in SIV and env-SHIV-infected macaques is highly dependent on the strength of antiviral immune responses [20,22,42,43]. Evidence from human trials also suggests a role of the immune system in determining the efficacy of drug treatment, as lower baseline viral RNA levels, a better status of the immune system, and certain MHC class II genotypes are predictive of a faster and/or more sustained response to HAART [44-49]. In HIV-1 infected humans, the reduction in viral RNA levels following the start of tenofovir therapy was larger and faster in treatment-naive patients than in treatment-experienced patients who generally had lower CD4+ T cell counts [50-52]. In the current study with macaques, RT-SHIV infection led to a reduction of CD4+ cell counts and percentages in peripheral blood, but an unexpected finding was that lower pre-therapy baseline values of percentage CD4+ T cells were associated with lower RNA levels, and a better early virologic response to tenofovir. While our study was not designed to unravel the mechanisms linking viremia and CD4+ T cell counts in peripheral blood, this observation mainly highlights that CD4+ T cell numbers in the peripheral blood of the macaques were not a very reliable marker of immunocompetence at this intermediate stage of RT-SHIV infection.

When RT-SHIV infected macaques were started on tenofovir, there was a rapid selection for viral mutants with a K70E RT mutation. The K70E RT mutants were subsequently replaced by K65R RT mutants. Similarly to observations with HIV-1 [53,54], when K70E and K65R RT-SHIV mutants were both detected in plasma, these 2 mutations were found on separate viral genomes. The K70E mutation has previously been described in HIV-1 after selection pressure with adefovir *in vitro *and *in vivo *[55,56]. The K70E mutation has only been described in a few cases of tenofovir-treated HIV-1 infected persons, although it is possible that a more systematic investigation of early samples following tenofovir therapy may reveal a higher frequency [54,57,58]. Because the regimen of these HIV-1 infected persons included also other RT inhibitors (e.g., abacavir and lamivudine; [54,57]), the detection of K70E virus in macaques during tenofovir monotherapy is the first evidence that tenofovir can select directly for this K70E mutation *in vivo*. In the current macaque study, it is possible that a higher precursor mutation frequency at codon 70 (but still below the 0.2% detection limit in the baseline samples) may have predisposed for selective outgrowth of first variants with the K70E mutation which, *in vitro*, confers only a relatively minor fitness cost and minimal resistance to tenofovir (ranging from no effect to ≤ 2-fold reduced susceptibility)[2,56,59]. These K70E mutants were then rapidly replaced by K65R mutants which in the absence of drug are more replication-impaired *in vitro *than K70E mutants, but in the presence of tenofovir could outgrow K70E mutants due to a slightly higher level (~4–5 fold) of *in vitro *resistance to tenofovir [56,60]. This replacement of variants based on replicative capacity would be similar to observations in lamivudine-treated patients, where differences in pre-therapy frequencies of codon 184 mutants also appear to explain the transient detection of M184I mutants, which are then replaced by the more fit M184V mutants [61]. While previous studies in macaques used usually higher tenofovir regimens (20–30 mg/kg subcutaneously SID; [20,62-64]), it is unclear if the 10 mg/kg tenofovir regimen of the current study may also have contributed to a transient outgrowth of K70E mutants that were subsequently, when intracellular drug levels built up to steady-state levels, replaced by the more resistant K65R mutants.

The emergence of K65R RT-SHIV mutants during tenofovir treatment was accompanied by an accumulation of other RT mutations, believed to be compensatory mutations that improve the replicative capacity of K65R virus. Many of these mutations have been described previously with or without K65R in HIV-1 infected persons receiving tenofovir-containing or other HAART regimens, and some (such as M41L) have been reported to contribute to a reduced virologic response to tenofovir when in combination with other RT mutations [5,8,14,34,59,65,66].

The clinical implications of the K70E and K65R RT-SHIV mutants were similar to those reported previously for K65R SIVmac251 mutants [19,20,22]. In the current RT-SHIV study, because treatment was initiated after 20 weeks of persistently high viremia, the immune system was already compromised. In such situation, for the majority of animals, CD8+ cell-mediated immune responses were not sufficient to assist in suppressing viremia to very low levels especially once the RT mutants emerged. Following the emergence of such viral mutants, plasma viremia of most animals increased and stabilized to a level on average 10-fold below pre-therapy baseline values, indicating some residual therapeutic benefit. This lower viremia may be due to a combination of several factors, including decreased replicative capacity of the K65R mutants (especially of the early mutants, prior to the accumulation of presumed compensatory mutations), some residual antiviral effects of the tenofovir regimen, and/or antiviral immune responses (see further). These findings are consistent with observations in tenofovir-treated humans, where the detection of K65R is not always associated with a virologic rebound [1,5], and the detection of the K65R mutation with a virologic rebound was most likely in patients with high baseline RNA levels and low CD4+ cell counts [3,6,9,67]. The findings of a ~10-fold reduced viremia in most K65R RT-SHIV infected animals during continued tenofovir monotherapy are reminiscent of observations in people who are infected with M184V mutant HIV-1, and for whom continuation of lamivudine monotherapy is associated with a ~2- to 4-fold reduction in viremia and clinical benefits [68-73]. In contrast, for some other drugs (such as nevirapine), the emergence of viral mutants has been associated with a rebound of viremia to pre-therapy levels [74].

While 11 of the 12 animals maintained persistent viremia and eventually developed disease, animal 30577 was an exception. Further research is needed to determine which host and/or viral factors (e.g., unique genotypes) may have been responsible for this different outcome, as animal 30577 was initially indistinguishable from the other animals based on our limited panel of pre- and early post-therapy markers (virus levels, CD4+ and CD8+ lymphocyte numbers, and emergence of RT mutations). Animal 30577 first had a rapid 47-fold reduction in viremia within 2 weeks of treatment but then had a 10-fold increase in viremia above nadir levels by 8 weeks of treatment, that was associated with the emergence of K70E followed by K65R viral mutants. Several human studies in which tenofovir-containing HAART regimens were initiated in antiretroviral-naïve patients used virologic criteria (< 2 log reduction in viral RNA by week 8; rebound of ≥ 0.5–1 log RNA copies/ml above nadir [6,11,67]) according to which this viral RNA pattern in animal 30577 would have been classified as a "virologic non-response" or "treatment failure", and tenofovir treatment would have been withdrawn. However, despite the initial viral RNA rebound in this macaque, continued tenofovir treatment led to a gradual decrease of viremia to undetectable levels after 8 months of therapy. Thus, our data suggest that further studies (including retrospective analysis of already available samples) are warranted to investigate the potential clinical benefits of continued tenofovir therapy in HIV-1 infected patients even after a partial rebound with the detection of K65R viral mutants, as it is possible that some individuals may eventually suppress viremia again [22].

The CD8+ cell depletion experiment demonstrated that the reduced viremia in tenofovir-treated animal 30577 was mediated largely by CD8+ cells, because removal of CD8+ cells caused a transient ~1 million-fold increase of K65R viremia, to peak levels similar to those observed during acute viremia with wild-type RT-SHIV (Fig. 3, 8). Because a stable tenofovir treatment regimen was continued during the CD8+ cell depletion experiment, this dramatic increase in K65R viremia, which was associated with a reduction in CD4+ T lymphocyte counts, indicates that in the absence of CD8+ cells, tenofovir treatment alone had insufficient inhibitory activity against K65R RT-SHIV, and this virus had good replicative capacity and virulence. This suggests that, at least when accompanied by other RT mutations, a potential attenuating effect of the K65R mutation on viral replicative capacity is by itself insufficient to explain the reduced viremia during tenofovir therapy. The demonstration of the direct causality between CD8+ cell-mediated immune responses and suppressed K65R viremia in tenofovir-treated animal 30577 is important, as it helps to explain observations in humans, where strong cell-mediated immune responses were associated with the maintenance of low-level viremia in HAART-treated individuals with drug-resistant HIV-1 [75-78].

Because the cM-T807 antibody depletes both the CD3+CD8+ T lymphocytes and CD3-CD8+ NK cells, the relative contribution of these 2 cell populations to the immune-mediated suppression of viremia in animal 30577 could not be determined in the current experiment. NK cells are also effector cells of antibody-dependent cellular cytotoxicity (ADCC). In an *in vitro *assay that measures antibody-dependent cell-mediated virus inhibition, plasma samples of animal 30577 and of tenofovir-treated SIVmac251-infected animals that similarly suppressed K65R viremia to undetectable levels were found to have high antiviral activity in the presence of PBMC effector cells [79]. In these long-term tenofovir-treated SIV or RT-SHIV infected animals with undetectable viremia, the antiviral immune responses must be unusually strong and broad, as immune-escape mutants have not emerged even after 6 to 11 years of tenofovir treatment ([20,43]; unpublished data). Further elucidation of these strong antiviral immune responses (including effector frequency, epitope recognition, etc.) may aid the development of novel immunotherapeutic strategies that combine the strengths of antiviral drugs and the immune system to indefinitely delay disease progression in HIV-infected persons.

Despite this important role of CD8+ cells, continued administration of tenofovir was still required to maintain maximal suppression of K65R viremia in animal 30577; in other words, both antiviral immune responses and tenofovir were needed. This is similar to previous observations in tenofovir-treated animals that were infected with K65R SIV mutants [20]. A recent report also demonstrated that both antiviral immune responses (induced by prior immunization) and short-term tenofovir administration were required to protect macaques against infection after intravenous inoculation with a high dose of a virulent K65R SIV isolate [80]. For RT-SHIV infected animal 30577, the viral rebound following withdrawal of tenofovir therapy was relatively slow (in comparison to the more rapid and dramatic rebound during CD8+ cell depletion), and consisted of K65R in PBMC-associated infectious virus, but K70E in plasma viral RNA. The reason for this discrepancy in mutations is unclear, but it is plausible that both sources of virus reflect distinct compartments, where differences in the relative role and strength of immunologic and pharmacologic factors may affect virus replication differently upon tenofovir withdrawal. As discussed previously, it is unclear whether the need of continued tenofovir treatment to achieve optimal inhibition of K65R virus replication is due to residual direct antiviral activity of the tenofovir regimen against these mutants (e.g., in antigen-presenting cells), and/or to potential immunomodulatory effects of tenofovir (such as priming of IL-12 secretion) that promote the generation and maintenance of strong antiviral immune responses [20,81]. It is important to remember that the effects of antiviral immune responses during drug therapy are not mutually exclusive of the effects of residual drug activity and/or reduced replicative capacity of mutant virus. In particular, even a partial inhibition of virus replication by the drug regimen, or a minor decrease in replicative capacity, can have a major impact on viremia if it provides more opportunity for effective antiviral immune responses to kill productively infected cells prior to the major viral burst. While our macaque studies modeled only the responses to tenofovir monotherapy, such considerations may be even more relevant for HAART-treated humans, as the combination of the K65R mutation with some other drug-selected mutations can further decrease viral replication fitness and affect drug susceptibility (including restored susceptibility) to tenofovir or other drugs of the HAART regimen, which may offer additional opportunities for antiviral activity.

## Conclusion

The current findings with RT-SHIV are consistent with but also extend previous observations on the K65R mutation in the SIV model [20,22,80], with the novel observation of the tenofovir-selected K70E mutation (which was not detected by population sequencing in tenofovir-treated SIVmac251-infected macaques). The observations in macaques suggest that for persons infected with K65R HIV-1, both immune-mediated and drug-dependent antiviral activities may play a role in controlling viremia, and that even in the presence of K65R virus, continuation of tenofovir treatment as part of HAART may be beneficial, particularly when assisted by antiviral immune responses. In other words, the detection of K65R may by itself not be a valid reason to withdraw tenofovir from the patient's regimen, unless more effective salvage regimens, which are also feasible in terms of cost, toxicity and compliance are available. While preliminary data already suggest the virologic benefits of including tenofovir in drug regimens for HIV-1 infected patients with K65R mutants [82,83], additional long-term studies are warranted to determine also the potential clinical benefits of continuing tenofovir therapy in the presence of K65R mutants. Such information is also relevant to develop treatment guidelines for resource-poor areas, where access to 2^nd ^or 3^rd ^line anti-HIV drugs may be limited, and regular monitoring of virus levels and drug resistance (such as K65R) is not always feasible. Simple treatment strategies for which decisions to alter the regimen would be less dependent on frequent monitoring of such laboratory parameters will be more practical and affordable, and can thus benefit the largest number of people.

## Methods

### Animals

All rhesus macaques (*Macaca mulatta*) were juvenile animals from the type D-retrovirus-free and SIV-free colony at the California National Primate Research Center (CNPRC), and were housed in accordance with American Association for Accreditation of Laboratory Animal Care standards, with strict adherence to the "Guide for the Care and Use of Laboratory Animals" [84]. For blood collections, animals were immobilized with 10 mg/kg intramuscular ketamine-HCL (Parke-Davis, Morris Plains, NJ, USA). Complete blood cell counts were measured by using an automated electronic cell counter (Baker 9000; Serono Baker Diagnostics); differential counts were determined manually.

### *In vitro *propagation of RT-SHIV

An infectious cell-free stock of RT-SHIV (which contains the RT of HIV-1 IIIB clone HXBc2; [24]) was prepared following transfection of CEMx174 cells by electroporation, as described previously [31]. Aliquots of cell-free supernatants were stored frozen at -130°C. This RT-SHIV stock had a titer of 10^5 ^50% tissue culture infectious doses (TCID_50_) per ml. This RT-SHIV stock had the T to C substitution at position 8 of the SIV tRNA primer binding site, which is necessary for rapid replication of RT-SHIV *in vivo *[85].

### Animal inoculation and *in vivo *passage of RT-SHIV

A first group of 3 animals (group A; Fig. 1) was inoculated intravenously with 1.0 ml of undiluted virus (i.e., 10^5 ^TCID_50_). Equal volumes of cryopreserved EDTA-anticoagulated plasma collected from the 3 animals at 2 weeks of infection were thawed, pooled and 0.6 ml was administered intravenously to 4 new animals (group B; Fig. 1). Similarly, EDTA-anticoagulated plasma collected from these 4 animals 2 weeks after virus inoculation was pooled and 0.6 ml was administered intravenously to another 5 animals (group C).

### Preparation and administration of tenofovir

Tenofovir (Gilead Sciences) was suspended in distilled water, dissolved by the addition of NaOH to a final pH of 7.0 and concentration of 60 mg/ml, filter sterilized (0.2 μm; Nalgene), and stored at 4°C. Starting at 20 or 21 weeks after RT-SHIV inoculation, tenofovir was administered subcutaneously into the back of the animal at a once daily dosage regimen of 10 mg/kg body weight, with dosage changes described in the results section. Animals were monitored regularly by chemistry panels and urinalysis to monitor for renal toxicity that occurs with prolonged high-dose tenofovir regimens, and make the necessary dosage adjustments [36].

### Administration of cM-T807

CD8+ cells were depleted using the previously described cM-T807 antibody [86,87]; a total of 20 mg/kg body weight was administered in 3 doses: 10 mg/kg subcutaneously on day 0, and 5 mg/kg intravenously 3 and 7 days later. No adverse effects were observed following cM-T807 administration.

### Quantitation of plasma viral RNA

Viral RNA levels in plasma were quantified using a real-time reverse transcription-polymerase chain reaction (RT-PCR) assay for SIV gag, described previously [88,89]. With the available plasma volumes, the sensitivity was 30 RNA copies/ml.

### Virus isolation

Infectious virus was isolated in cultures of peripheral blood mononuclear cells (PBMC) with CEMx174 cells and subsequent p27 core antigen measurement, according to methods previously described [90]. Levels of infectious virus in PBMC and plasma were determined by a limiting dilution assay [90].

### Drug susceptibility assays

Phenotypic drug susceptibilities of RT-SHIV isolates were characterized by a previously described assay based on a dose-dependent reduction of viral infectivity [19,91].

### Sequence analysis of RT-encoding region

Infected CEMx174 and PBMC co-cultures were harvested as soon as culture supernatants were positive by antigen capture ELISA. Genomic DNA was extracted and used for nested PCR; amplicons were purified with a PCR purification kit (QIAGEN) and used for DNA sequencing according to methods and with primers described previously [25,31]. This method can detect the presence of a 20% subpopulation.

### Real-time polymerase chain reaction (PCR) for sensitive detection of K65R and K70E in plasma RT-SHIV RNA

Sensitive testing for the K65R and K70E mutations was performed using real-time PCR-based methodologies as described previously [92]. Briefly, a 763 nucleotide template of RT-SHIV (n.t. 58 to 821 in RT) was first amplified by RT-PCR using the HIV-1 RT primer RTP-REV (5'-ATC CCT GCA TAA ATC TGA CTT GC) for the reverse transcriptase step followed by addition of forward primer RTP-F2 (5'-AAA GTT AAA CAA TGG CCA TTG ACA G) for PCR amplification.

The real-time PCR reactions were performed in duplicate using 2 μl of the RT-PCR products for both the total virus copy and mutation-specific tests. Total SHIV RT templates were detected with the primers ComFWD and ComREV, which span n.t. 258–420 in RT, along with the common probe 1. The reactions for detecting the K65R mutation involved the mutation-specific primer HIV-RT 65R.FWD with the primer 65R.REV and FAM-labeled probes mixture, 1P (80%) and 2P (20%). The K70E test used the mutation-specific primer 70E.REV with the primer 70.FWD and probe 70.2P. Differences in total copy and mutation-specific amplification curves (ΔCT) of ≤9 or ≤ 10 cycles indicated the presence of 65R and 70E, respectively. These assay cutoffs allowed mutant viruses to be detected at frequencies ≥ 0.4%.

### Mutation linkage analysis

To evaluate the interplay of the mutations during emergence, the positive mutation-specific amplicons were directly sequenced using the primers 65-118SEQ.1R (5'-CTA GGT ATG GTA AAT GCA GTA TAC TTC CT) and RT-SEQ.2F (5'-AAG GAA GGG AAA ATT TCA AAA ATT GGG CC) for the K65R amplicon and K70E amplicon, respectively. The overlapping amplicon sequences allowed for visualization of the adjacent codons.

### Detection of SIV-specific immune responses

The ELISA to detect SIV-specific immunoglobulin G (IgG) in plasma samples was performed as described previously [22].

### Lymphocyte phenotyping

Initially, 3-color flow cytometry techniques were used to detect CD3, CD4, CD8 and CD20 with fluorochrome-conjugated antibodies described previously [93,94]. Starting at approximately 145 weeks of infection, 4-color flow cytometry techniques were used, consisting of a single tube containing antibodies to CD3, CD4, CD8 and CD20, as described previously [20]. CD4+ T lymphocytes and CD8+ T lymphocytes were defined as CD3+CD4+ and CD3+CD8+ lymphocyte populations, respectively. B lymphocytes were defined as CD3-CD20+ lymphocytes. NK cells were defined as CD3-CD8+ lymphocytes. During the CD8+ cell depletion experiment, the anti-CD8 antibody was replaced by the DK25 clone (DAKO, Carpinteria, California) conjugated to FITC (and combined with anti-CD3-PerCP, anti-CD4-PE and anti-CD20-APC as decribed previously [20]).

### Genetic assessment of MHC class I alleles

DNA extracted from lymphoid cells (with QIAamp^® ^DNA mini kit, QIAgen, Valencia, CA) was used to screen for the presence of the major histocompatibility complex (MHC) class I alleles Mamu-A*01 and Mamu-B*01, using a PCR-based technique [95,96]. The frequency of Mamu-A*01 and Mamu-B*01 alleles in the CNPRC rhesus macaque colony is approximately 25%.

### Criteria for euthanasia and animal necropsies

Euthanasia of animals with simian AIDS was performed when clinical observations indicated a severe life-threatening situation for the animal, as described previously [22]. A complete necropsy with a routine histopathologic examination of tissues was performed. Tissues were fixed in 10% buffered formalin, embedded in paraffin, sectioned at 6 μm, stained with hematoxylin and eosin, and examined by light microscopy.

### Statistical analysis

Statistical analyses were performed with Prism Version 4.0 and Instat Version 3.0a (GraphPad Software Inc. San Diego, CA). All statistical analyses of viral RNA levels in plasma were performed after log-transformation of the values.

## Competing interests

This study was partially supported by Gilead Sciences.

## Authors' contributions

KKAVR was responsible for the overall design of the study, sample processing, data analysis and preparation of the first draft of the manuscript. JAJ, JL and WH contributed all real-time PCR data including data analysis and interpretation. EJB, RPS and TBM performed DNA sequence analysis, virus isolations and data entry. NB assisted with pharmacokinetic analyses and data interpretation. MLM, NCP, and TWN assisted with the study design and data interpretation. All authors were involved in revising the manuscript draft and approved the final manuscript.
